# Drug-drug interactions with oral anticoagulants: information consistency assessment of three commonly used online drug interactions databases in Switzerland

**DOI:** 10.3389/fphar.2024.1332147

**Published:** 2024-04-02

**Authors:** Claire Coumau, Frederic Gaspar, Jean Terrier, Angela Schulthess-Lisibach, Monika Lutters, Marie-Annick Le Pogam, Chantal Csajka

**Affiliations:** ^1^ Centre for Research and Innovation in Clinical Pharmaceutical Sciences, University Hospital and University of Lausanne, Lausanne, Switzerland; ^2^ School of Pharmaceutical Sciences, University of Geneva, Geneva, Switzerland; ^3^ Division of General Internal Medicine, Geneva University Hospitals, Geneva, Switzerland; ^4^ Geneva Platelet Group, Faculty of Medicine, University of Geneva, Geneva, Switzerland; ^5^ Clinical Pharmacology and Toxicology Division, Anesthesiology, Pharmacology, Intensive Care and Emergency Medicine Department, Geneva University Hospitals, Geneva, Switzerland; ^6^ Institute of Primary Healthcare (BIHAM), Faculty of Medicine, University of Bern, Bern, Switzerland; ^7^ Clinical Pharmacy, Cantonal Hospital Aarau, Aarau, Switzerland; ^8^ Department of Epidemiology and Health Systems, Center for Primary Care and Public Health (Unisanté), University of Lausanne, Lausanne, Switzerland; ^9^ Institute of Pharmaceutical Sciences of Western Switzerland, University of Geneva, University of Lausanne, Lausanne, Switzerland

**Keywords:** drug-drug interaction databases, oral anticoagulant, consistency, discrepancies, risk classification, drug information, direct oral anticoagulant,, vitamin K antagonist

## Abstract

**Background:** Toxicity or treatment failure related to drug-drug interactions (DDIs) are known to significantly affect morbidity and hospitalization rates. Despite the availability of numerous databases for DDIs identification and management, their information often differs. Oral anticoagulants are deemed at risk of DDIs and a leading cause of adverse drug events, most of which being preventable. Although many databases include DDIs involving anticoagulants, none are specialized in them.

**Aim and method:** This study aims to compare the DDIs information content of four direct oral anticoagulants and two vitamin K antagonists in three major DDI databases used in Switzerland: Lexi-Interact, Pharmavista, and MediQ. It evaluates the consistency of DDIs information in terms of differences in severity rating systems, mechanism of interaction, extraction and documentation processes and transparency.

**Results:** This study revealed 2’496 DDIs for the six anticoagulants, with discrepant risk classifications. Only 13.2% of DDIs were common to all three databases. Overall concordance in risk classification (high, moderate, and low risk) was slight (Fleiss’ kappa = 0.131), while high-risk DDIs demonstrated a fair agreement (Fleiss’ kappa = 0.398). The nature and the mechanism of the DDIs were more consistent across databases. Qualitative assessments highlighted differences in the documentation process and transparency, and similarities for availability of risk classification and references.

**Discussion:** This study highlights the discrepancies between three commonly used DDI databases and the inconsistency in how terminology is standardised and incorporated when classifying these DDIs. It also highlights the need for the creation of specialised tools for anticoagulant-related interactions.

## 1 Introduction

Drug-drug interactions (DDIs) are of significant concern in clinical practice, especially for drug classes at particular risk (McDonnell and Jacobs, 2002; Becker et al., 2007). Oral anticoagulants are particularly noteworthy, with several studies identifying them as a leading cause of adverse drug events (ADEs), resulting in increased morbidity, mortality and hospitalization (Budnitz et al., 2011; Jolivot et al., 2014; Shehab et al., 2016; Oscanoa et al., 2017; Ayalew et al., 2019). It has been reported that at least half of ADEs associated with oral anticoagulants, including DDIs, can be prevented (Sennesael et al., 2018; Spada et al., 2020). Vitamins K antagonists (VKAs) were the first oral anticoagulants introduced on the market and are still widely prescribed despite their narrow therapeutic range that requires constant monitoring. A large body of quality evidence shows that VKAs are subject to numerous drug and food interactions (Penning-van Beest et al., 2001; Holbrook et al., 2005; Violi et al., 2016). The introduction of direct oral anticoagulants (DOACs) offered an interesting alternative to VKAs. DOACs exhibit a more predictable dose-concentration-response relationship, have a broader therapeutic index, and do not require routine monitoring for dosage adjustment (Ageno et al., 2012; Cabral, 2013; Nutescu et al., 2016). While this therapeutic class was initially reported to be less prone to DDIs (Di Minno et al., 2017), recent findings showed that interactions involving DOACs are associated with significant risks of ADEs (Lee et al., 2020; Li et al., 2020; Terrier et al., 2021). The lack of direct monitoring complicates DDI management.

To address DDIs in daily clinical practice, numerous computer decisions support tools and online databases have been developed (Clauson et al., 2007; Roblek et al., 2015). A primary concern with these electronic DDIs databases is the overwhelming number of DDIs leading to alerts, many of which have minimal clinical relevance. This leads to an “over-alerting” effect on prescribers, diminishing their vigilance and obscuring the true significance of the DDIs (Glassman et al., 2002; Paterno et al., 2009; Smithburger et al., 2011; Roblek et al., 2015). Furthermore, the information across these databases often lacks consistency. They frequently present conflicting advice, varying severity classifications, and significant differences in sensitivity and specificity for detecting DDIs (Abarca et al., 2006; Clauson et al., 2007; Vonbach et al., 2008; Sweidan et al., 2009; Reis and Cassiani, 2010; Kongsholm et al., 2015; Roblek et al., 2015). Understanding the risk associated with DDIs involving oral anticoagulants is of major importance to minimize the risk of hemorrhages or thromboses (Ufer, 2005; Vazquez, 2018; Hanigan et al., 2020; Li et al., 2020; Gronich et al., 2021; Holm et al., 2021; Grymonprez et al., 2023). While several databases list DDIs associated with oral anticoagulants, none specialize exclusively in this category. The aim of this cross-sectional study was to compare the DDIs information content of four direct oral anticoagulants (DOACs) apixaban, rivaroxaban, edoxaban and dabigatran and two vitamin K antagonists (VKAs) acenocoumarol and phenprocoumon in the three predominant online databases used in clinical practice in Switzerland: Lexi-Interact, Pharmavista and MediQ. The specific aims were to assess the consistency of the DDI information provided and to evaluate the information content, transparency of ownership, sources of funding, level of documentation and staff qualifications associated with each of these three databases.

## 2 Materials and methods

### 2.1 Study design, databases and drugs selection

We conducted a cross-sectional study with the aim of comparing oral anticoagulant DDIs among three well-established databases commonly used in Switzerland, namely Lexi-Interact, Pharmavista and MediQ. Pharmavista is known to be widely used in community pharmacies, and both Lexi-Interact and MediQ are favored in hospital settings in the French (Vonbach et al., 2008) and German parts (Frölich et al., 2011; Guzek et al., 2011; Haueis et al., 2011; Zorina et al., 2012; Zorina et al., 2013), respectively. The study design involved the assessment of various parameters, including the number and nature of DDIs, risk classification, interaction type and mechanism, as well as information available on each database’s website. Our study focused on the four DOACs available in Switzerland: edoxaban, rivaroxaban, apixaban, and dabigatran. Additionally, we examined the two VKAs marketed in Switzerland: acenocoumarol which is the VKA of choice in French-speaking Switzerland, and phenprocoumon, which is widely prescribed in the German-speaking areas (Niederer et al., 2001; Bochatay et al., 2016).

### 2.2 Data extraction

Data extraction covered the period from July 2021 to January 2022. All available interacting compounds were considered, including food, herbal products, dietary supplements, tobacco, and ethanol. The interaction lists obtained from the three DDI databases were compiled in a descriptive table summarizing the following items: (1) the international non-proprietary name (INN), (2) the therapeutic group according to the ATC (Anatomical Therapeutic Chemical) classification), (3) the type of interaction [i.e. pharmacokinetic (PK) and/or pharmacodynamic (PD)], and (4) details of the interaction mechanism. In parallel, a qualitative assessment of the quality and transparency of ownership, funding, information, classifications, staff training and underlying documentation of the DDIs database was performed by retrieving the following information from the websites: formal ownership and funding, information on potential conflict of interest, presence of a DDI interaction classification, availability of a procedure for categorizing the severity of DDIs, definition and procedure for categorizing the level of evidence for DDIs, availability of scientific references, staff qualifications, and information on database updates. Secondly, we sought to recover the missing information by contacting the DDIs database managers.

### 2.3 Data analysis

The sources consulted were compared in terms of similarities in the number and in the risk classification of DDIs detected by each database. The number of DDIs in each database, the number of similar DDIs in two and in the three databases, and the proportion of DDIs considering the total number of retrieved DDIs in all three databases were assessed. Information on the nature of the DDIs (PK and PD) and the interaction mechanism described in each database were also compared. A further analysis was performed by comparing the number of DDIs classified as “high-risk” by one, two, or all three databases, together with a description of the molecule names classified similarly or divergently among them. Hypotheses underlying the potential differences in classification were also made for the discrepant DDIs. Quantitative results were reported as means and proportions as appropriate.

Given that each DDI database employs a different rating system for assessing the severity of interactions (four risk categories for MediQ, five for Lexi-Interact, and six for Pharmavista), we constructed an equivalent risk-level classification, which is provided in Table 1.

**TABLE 1:** Drug-drug interaction severity rating systems and equivalence of risk levels between Lexi-Interact, Pharmavista, and MediQ databases.

Lexi-interact	Pharmavista	MediQ	Equivalent risk level
X: Avoid combination	1: Contraindicated	3: High interaction	High
	2: Contraindicated (as a precaution)		
D: Consider therapy modification	3: Adapt therapy	2: Average interaction	Moderate
C: Monitor therapy	4: Monitoring if risk factors		
B: No action needed	5: Monitoring (as a precaution)	1: Low interaction	Low
A: No known interaction	6: No action required	?: This combination has not been described yet	

The overall concordance in the equivalent risk-level classification provided by the three DDI databases was evaluated using the Fleiss’ kappa and Gwet’s AC1 coefficient, while Cohen’s kappa coefficient was used to determine the pairwise classification concordance among two lists. The following degrees of agreement for kappa coefficients suggested by Landis and Koch (Landis and Koch, 1977) were used: a kappa value > 0.80 was considered “almost perfect,” 0.61–0.80, “substantial,” 0.41–0.60, “moderate,” 0.21–0.40, “fair,” 0.00–0.20, “slight” and < 0.00, “poor” (Landis and Koch, 1977). A *p*-value associated with the kappa coefficient was also calculated, with a *p*-value <0.05 indicating statistically significant agreement. Data management and analyses were performed with STATA (StataCorp. 2021. Stata Statistical Software: Release 17. College Station, TX: StataCorp LLC). Inter-rater agreement for nominal categorial variables (Cohen’s kappa, Fleiss’ kappa, and Gwet’s AC1 with 95% CI) was calculated with a STATA specific plug-in.

### 2.4 Qualitative assessment of available database website information

We conducted a qualitative analysis of the information available on each database’s website using a questionnaire derived from the systematic review by Kongholm et al. (Kongsholm et al., 2015). We then assessed whether specific information was present or absent across the three DDI databases.

## 3 Results

### 3.1 Overall drug interactions

In total, 2′496 DDIs were retrieved from the three databases for the six oral anticoagulants studied: 335 DDIs for apixaban, 316 for dabigatran, 355 for rivaroxaban, 287 for edoxaban, 592 for acenocoumarol and 613 for phenprocoumon. Lexi-Interact, Pharmavista and MediQ provided, respectively 76.6% (*n* = 1′912), 41.3% (*n* = 1′031) and 31.4% (*n* = 783) of total DDIs. Overall, only 13.2% (*n* = 330) of DDIs were common to all three databases, whereas 23.0% (*n* = 571) were common to two databases and 63.8% (*n* = 1′594) were present in only one source. Table 2 presents the number and proportion of DDIs for each anticoagulant.

**TABLE 2:** Comparison of total and high, moderate and low-risk drug-drug interactions (DDIs) identified by one or more DDIs databases.

	Lexi-interact n (%)	MediQ n (%)	Pharmavista n (%)	Lexi-interact ∩ MediQ n (%)	Lexi-interact ∩ pharmavista n (%)	Pharmavista ∩ MediQ n (%)	Lexi-interact ∩ pharmavista ∩ MediQ n (%)
Overall DDIs							
Apixaban (N = 335)	170 (50.7)	54 (16.1)	10 (3.0)	14 (4.2)	48 (14.3)	2 (0.60)	37 (11.0)
Rivaroxaban (N = 355)	158 (44.5)	80 (22.5)	12 (3.4)	10 (2.8)	47 (13.2)	3 (0.8)	45 (12.7)
Edoxaban (N = 287)	162 (56.8)	29 (10.1)	10 (3.5)	9 (3.2)	48 (16.8)	2 (0.70)	24 (8.4)
Dabigatran (N = 316)	130 (41.1)	34 (10.8)	37 (11.7)	6 (1.9)	62 (19.6)	11 (3.5)	36 (11.4)
Acenocoumarol (N = 592)	246 (41.6)	33 (5.6)	68 (11.5)	16 (2.7)	121 (20.4)	23 (3.9)	85 (14.4)
Phenprocoumon (N = 613)	216 (35.2)	72 (11.7)	73 (11.9)	25 (4.1)	94 (15.3)	30 (4.9)	103 (16.8)
High-risk DDI classification							
Apixaban (*N*_*high*_ = 54)	16 (29.6)	3 (5.6)	22 (40.7)	2 (3.7)	11 (20.4)	0 (0.0)	0 (0.0)
Rivaroxaban (*N*_*high*_ = 57)	17 (29.8)	1 (1.8)	15 (26.3)	2 (3.5)	13 (22.8)	4 (7.0)	5 (8.8)
Edoxaban (*N*_*high*_ = 34)	8 (23.5)	3 (8.8)	9 (26.5)	2 (5.9)	9 (26.5)	2 (5.9)	1 (2.9)
Dabigatran (*N*_*high*_ = 62)	15 (24.2)	4 (6.5)	23 (37.1)	0 (0.0)	11 (17.7)	4 (6.5)	5 (8.1)
Acenocoumarol (*N*_*high*_ = 33)	6 (18.2)	3 (9.1)	22 (66.7)	0 (0.0)	2 (6.1)	0 (0.0)	0 (0.0)
Phenprocoumon (*N*_*high*_ = 35)	6 (17.1)	5 (14.3)	18 (51.4)	0 (0.0)	2 (5.7)	3 (8.6)	1 (2.9)
Moderate-risk DDI classification							
Apixaban (*N*_*moderate*_ = 217)	145 (66.8)	15 (6.9)	5 (2.3)	13 (6.0)	27 (12.4)	4 (1.8)	8 (3.7)
Rivaroxaban (*N*_*moderate*_ = 201)	134 (66.7)	13 (6.5)	9 (4.5)	3 (1.5)	30 (14.9)	2 (1.0)	10 (5.0)
Edoxaban (*N*_*moderate*_ = 230)	182 (79.1)	1 (0.4)	4 (1.7)	6 (2.6)	28 (12.2)	0 (0.0)	9 (3.9)
Dabigatran (*N*_*moderate*_ = 207)	156 (75.4)	12 (5.8)	9 (4.3)	19 (9.2)	9 (4.3)	0 (0.0)	2 (1.0)
Acenocoumarol (*N*_*moderate*_ = 497)	323 (65.0)	10 (2.0)	34 (6.8)	21 (4.2)	76 (15.3)	8 (1.6)	25 (5.0)
Phenprocoumon (*N*_*moderate*_ = 481)	279 (58.0)	20 (4.2)	38 (7.9)	42 (8.7)	64 (13.3)	6 (1.2)	32 (6.7)
Low-risk DDI classification							
Apixaban (*N*_*low*_ = 118)	44 (37.3)	51 (43.2)	12 (10.2)	3 (2.5)	0 (0.0)	8 (6.8)	0 (0.0)
Rivaroxaban (*N*_*low*_ = 152)	44 (28.9)	87 (57.2)	10 (6.6)	2 (1.3)	0 (0.0)	9 (5.9)	0 (0.0)
Edoxaban (*N*_*low*_ = 58)	0 (0.0)	34 (58.6)	18 (31.0)	0 (0.0)	0 (0.0)	6 (10.3)	0 (0.0)
Dabigatran (*N*_*low*_ = 135)	15 (11.1)	36 (26.7)	78 (57.8)	1 (0.7)	1 (0.7)	4 (3.0)	0 (0.0)
Acenocoumarol (*N*_*low*_ = 196)	10 (5.1)	56 (28.6)	94 (48.0)	0 (0.0)	2 (1.0)	31 (15.8)	3 (1.5)
Phenprocoumon (*N*_*low*_ = 230)	5 (2.2)	86 (37.4)	102 (44.3)	3 (1.3)	2 (0.9)	30 (13.0)	2 (0.9)

Abbreviations: N = total number of DDI, across all databases; N_high_ = total number of DDI, at high-risk across all databases; N_moderate_ = total number of DDI, at moderate-risk across all databases; N_low_ = total number of DDI, at low-risk across all databases; ∩ = DDI, common to two or three databases.

**FIGURE 1:**
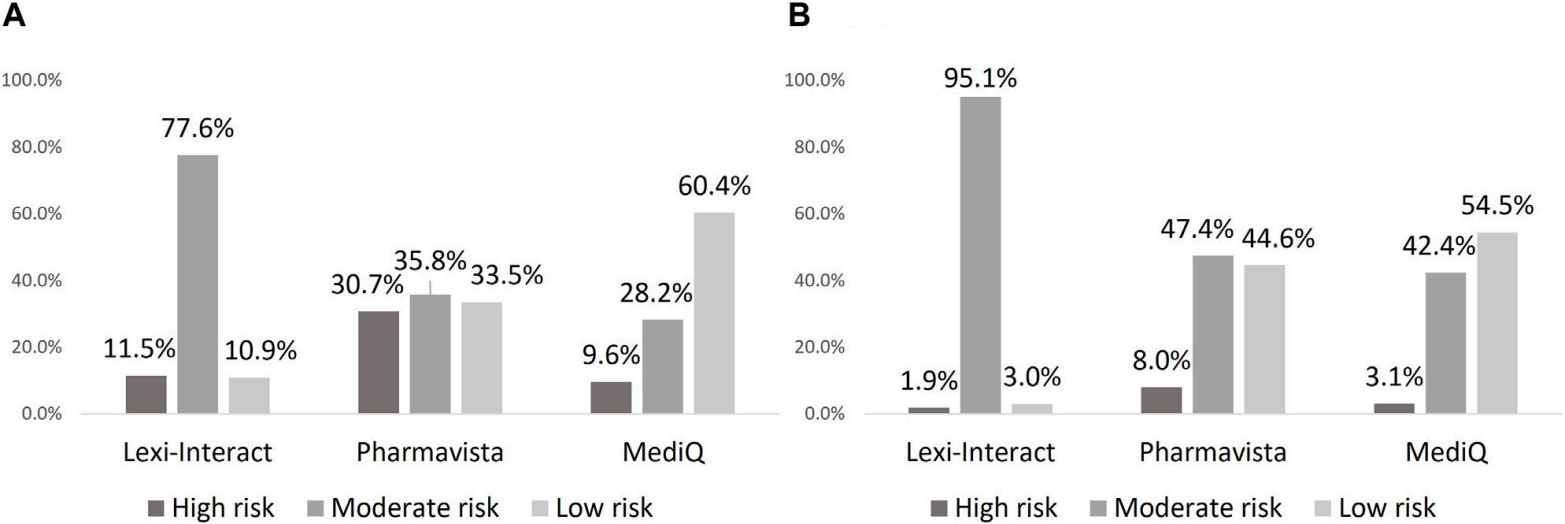
Proportion of drug-drug interaction according to severity in each database: DOAC **(A)**, VKA **(B)**.

The classification of the DDI risk level differed between the three databases. Lexi-Interact classified DDIs as moderate in a larger proportion of cases than Pharmavista, whereas the latter showed the highest proportion of high-risk DDI. On the other hand, MediQ showed the highest rate of low risk DDI (Figure 1). Similar proportions were observed between DOACs and VKAs. The description of DDI classification detected by the 3 databases, each using its own level of classification is presented in Supplementary Table SA.

Our analysis indicates that 55.4% (i.e., 63.2% for DOACs and 47.1% for VKAs) of the discrepancies are related to the classification of DDIs by a single DDI database. For DDIs classified by at least two of the three DDI databases, the majority of DDI discrepancies are congruent on the nature of the DDI, namely PK, PD, or unknown mechanisms, with only 16 discrepancies (4 for DOAC and 12 for VKA) in at least two of the three databases. For tipranavir (with apixaban or acenocoumarol), azithromycine (with apixaban), carvedilol (with rivaroxaban), levetiracetam (with rivaroxaban) and fusidic acid (with phenprocoumon) (*n* = 5), the mismatch concerned the PK or PD etiology of the interaction mechanism; for ceritinib, chloramphenicol and ifosfamide with phenprocoumon (*n* = 3), the discrepancy concerned the cytochrome linked to PK interaction, and the remaining 8 discrepancies were due to unidentified interaction mechanism in one database, while others suggested PK or PD causes. These discrepancies pertain to the following drugs: celecoxib, disulfiram, doxycycline, tamoxifen (*n* = 2), quinidine, eplerenone, and oxacillin in interaction with VKA. The detailed description of the divergent DDIs mechanism of the 16 drugs across the three databases is summarized in Supplementary Table SB.

### 3.2 High-risk DDIs classification comparison

**FIGURE 2:**
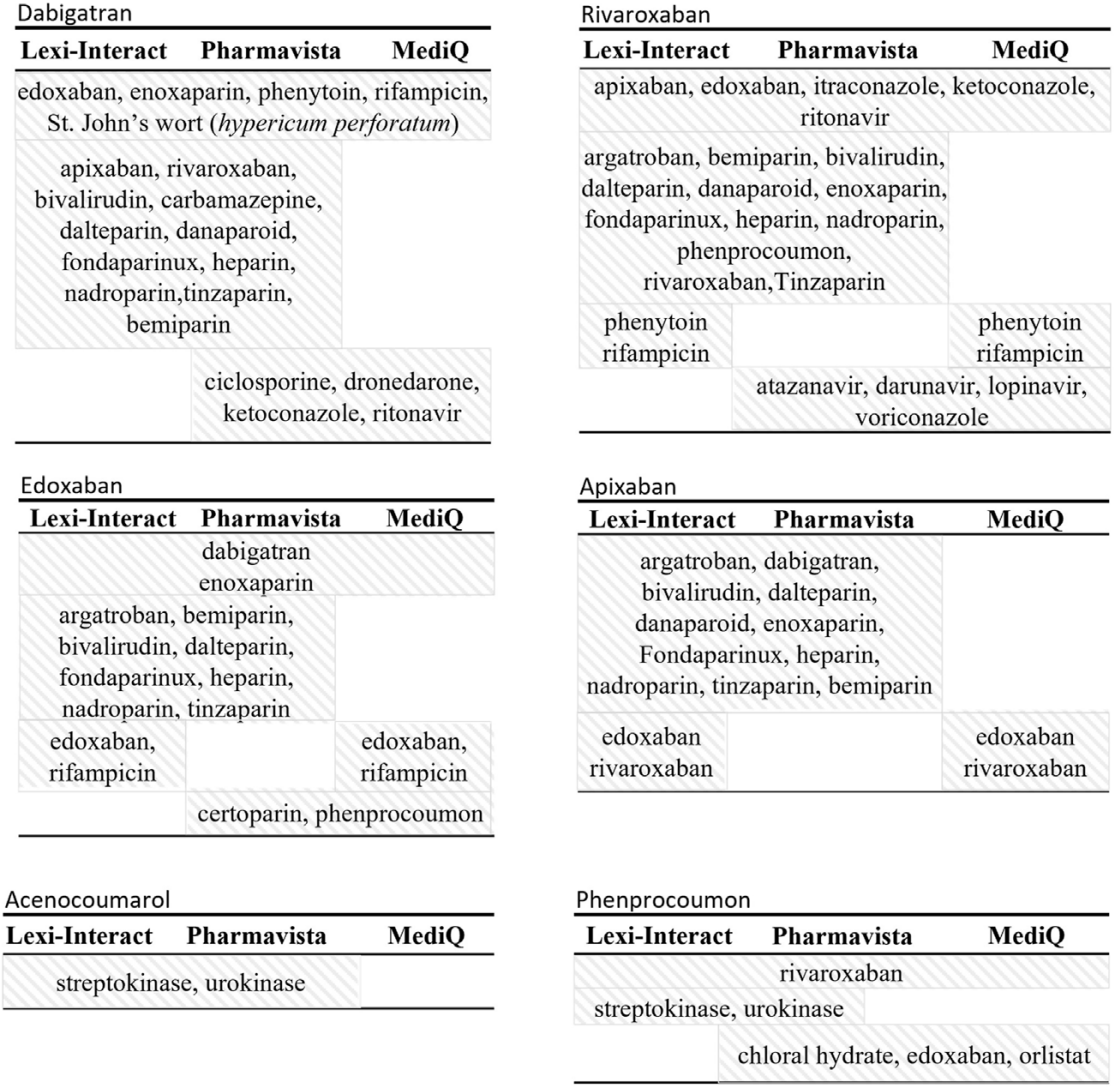
Description of molecule names reported as high-risk drug-drug interaction by either two or all three databases.

**FIGURE 3:**
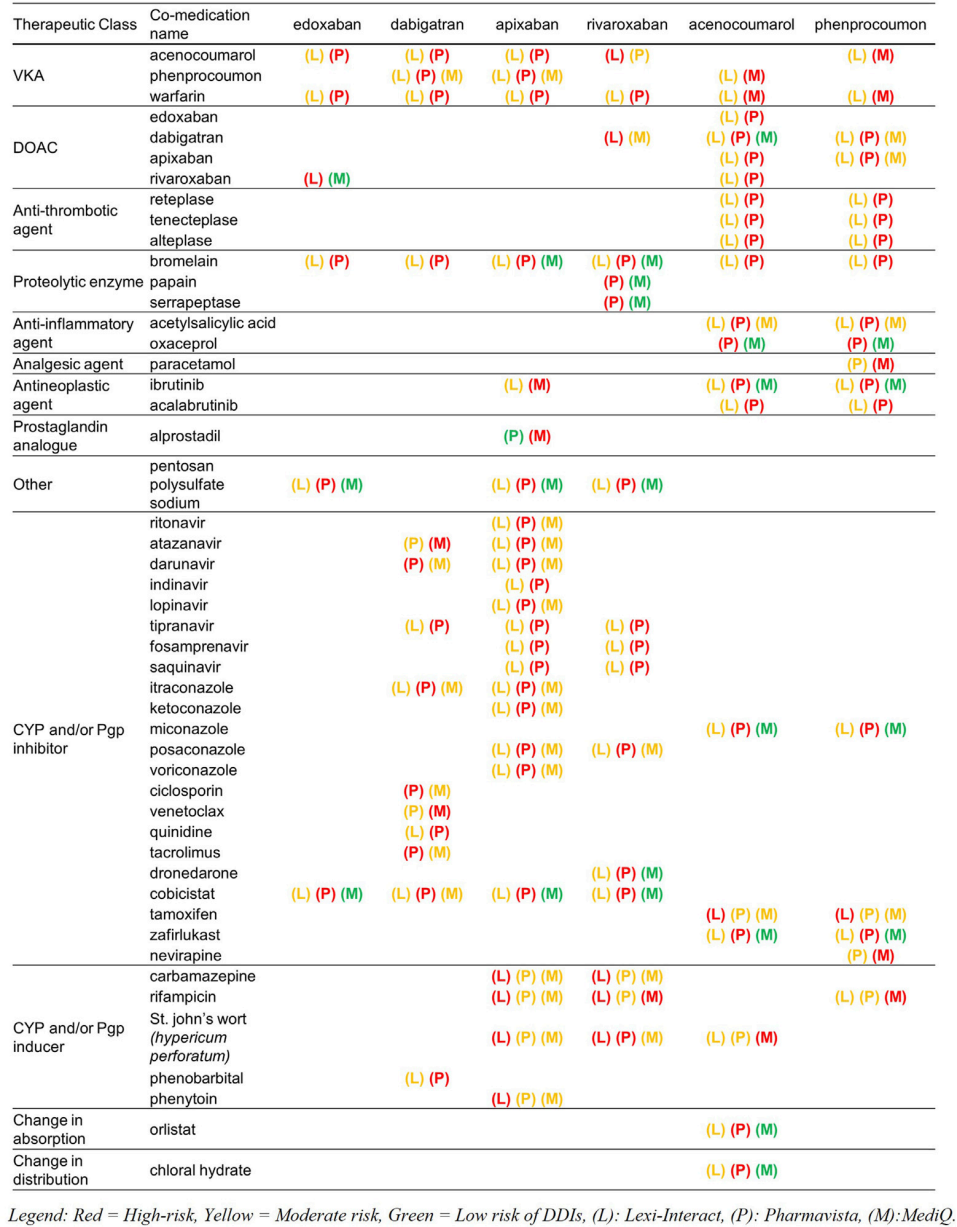
List of high-risk drug-drug interactions (DDIs) according to at least one database, with their classifications across the other two databases. CYP, Cytochrome P450; Pgp, P-glycoprotein.

A total of 207 and 68 high-risk DDIs for DOAC and VKA were retrieved, respectively (Table 2). Molecules that were classified as having a high-risk of DDI by the three databases are other anticoagulants with an additive effect on hemostasis (PD interaction) and strong inhibitors or inducers of the cytochromes and/or Pgp involved in the metabolism of the DOAC and the VKA (i.e., phenytoin, ketoconazole, itraconazole, rifampicin, St John’s wort (Hyperium Perforatum), resulting in a change in drug exposure and, as a consequence, a lack of efficacy or toxicity. Figure 2 presents the molecules classified as high-risk in two or three databases. A listing of high-risk DDIs with varying classifications across the three databases is presented in Figure 3.

### 3.3 Agreement between risk classification among the DDI databases

The Fleiss’ kappa and the Gwet’s AC1 values for comparing the three databases was 0.131 [0.080, 0.183, *p* < 0.001], and 0.324 [0.276, 0.372, *p* < 0.001] which corresponds to a slight and a fair level of agreement, respectively, according to Landis and Koch (Landis and Koch, 1977). The classification comparison for high-risk DDIs showed a higher Fleiss’ kappa value of 0.398 (*p* < 0.05), which represents a fair level of agreement (Landis and Koch, 1977). Table 3 summarizes the agreement for low, moderate, and high-risk of DDI.

**TABLE 3:** Agreement among three drug-drug interaction (DDI) databases.

Category of DDI	Fleiss kappa	*p*-value	Level of agreement
High-risk	0.398	<0.05	Fair
Moderate risk	0.047	0.043	Slight
Low risk	0.051	0.031	Slight
**Combined**	**0.131**	**< 0.05**	**Slight**

The values in bold correspond to the values obtained for the 3 risk classifications combined.

The Cohen’s kappa values showed a slight classification agreement between all databases pairs. A more consistent agreement was found between Pharmavista and MediQ with a Cohen’s kappa coefficient of 0.277 [95% CI 0.198, 0.356, *p* < 0.05] which corresponds to a fair level of agreement (Landis and Koch, 1977). Cohen’s kappa coefficient between Lexi-Interact and Pharmavista and between Lexi-Interact and MediQ was 0.177 [95% CI 0.137, 0.218, *p* < 0.05] and 0.140 [95% CI 0.079, 0.201, *p* < 0.05] respectively, which corresponds to a slight level of agreement.

### 3.4 Qualitative assessment of available database website information

Table 4 summarizes results of the qualitative assessment of the DDI databases. Information details concerning the three drug-drug interaction databases is available in Supplementary Table SC. All three databases offered severity classification, but a standard operating procedure for the categorization of DDI severity was only provided by Lexi-Interact. Additionally, only Lexi-Interact defined a level of evidence, also including information on the procedure used for categorizing the level of evidence. References were available in all databases.

**TABLE 4:** Qualitative assessment of the information available on each database website.

Database	Lexi-interact	Pharmavista	MediQ
Ownership			
Formal ownership	Wolthers Kluwer Health (listed information service company)	HCI solutions SA	The Aargau Psychiatric society
Funding	Yes	No	No
Information on potential conflicts of interest	No	No	No
Extraction and classification of interactions			
Explanation of the data extraction process	No^a^	Yes	No
Presence of a severity classification	Yes	Yes	Yes
Standard operating procedure (SOP) for categorization of severity classification	Yes	No	No
Level of evidence defined	Yes	No	No
Standard operating procedure for categorization of level of evidence	Yes	No	No
References available for each interaction	Yes	Yes	Yes
Staff qualifications			
Description of staff (who are they? What are their qualifications ?)	No^a^	No^a^	Yes
Other			
Disponibility	Yes [Subscription (by paying)]	Yes [Subscription (by paying)]	Yes [Subscription (by paying)]
Information on database updates	No^a^	Yes	Yes

^a^ Additional information provided by the drug-drug interactions (DDIs) manager upon request.

Upon request, Lexi-Interact provided a comprehensive overview of the data extraction process and supplied detailed information, including author names, their respective professional affiliations, and collaborating faculties for each medical specialty. Updates of its content are made by a continual review of new drug approvals, revisions to product labeling, and surveillance of published literature. Pharmavista outlined the qualifications of the staff responsible for reviewing DDIs. It did not provide the procedures of classification of the DDI severity to the reader, but these processes seemed available for their staff in their “working instructions.” The classification was based on PK and PD considerations, with reference to professional information, published literature, with regular updates performed by an experienced team of physicians and pharmacists. We did not have any further information by MediQ.

## 4 Discussion

Our research highlighted notable inconsistencies among three frequently used DDI databases concerning oral anticoagulants. Only a minor proportion of DDIs were consistently captured and categorized at the same risk level, raising particular concerns for the management of DDIs in clinical practice.

Our findings indicate a poor concordance rate of 23% between two and 13% among all three DDI databases, which are in line with previous reported studies examining other databases, drug classes or patient population (Vitry, 2007; Roblek et al., 2015; Shariff et al., 2021; Hecker et al., 2022; Kontsioti et al., 2022). For instance, a study conducted by Vonbach et al. reported a concordance of only 11% in DDIs involving the 100 most frequently used drugs at the cantonal Hospital of Baden among five databases, including Pharmavista and Lexi-Interact (Vonbach et al., 2008). Our results show Lexi-Interact provides the highest number of DDIs, which is consistent with a systematic review assessing the accuracy, completeness and user-friendliness of eight databases including Lexi-Interact, Pharmavista, and MediQ, that indeed reported that Lexi-Interact outperformed other databases in terms of completeness (Pauly et al., 2015).

Discrepancies between databases were mostly related to differences in the number of DDIs and their risk classification, rather than in the underlying mechanism of these interactions. The largest proportion of Lexi-Interact DDIs were classified as “moderate-risk,” whereas it was “low-risk” for MediQ, and Pharmavista listed approximately three times as many high-risk DDIs compared to the other databases. Lexi-Interact designates a DDI as “high-risk” only if it poses a life-threatening risk to the patient, providing a good sensitivity for drugs to absolutely avoid. Pharmavista seems more conservative in its way of classifying “high-risk” DDIs than Lexi-interact, and of the three databases, MediQ appears less concerned about DDIs, specially related to DOACs and to VKAs to a slightly lesser extent.

The Gwet’s AC1 coefficient was computed due to the imbalanced distribution among the observed categories. Similar results were obtained between Fleiss’ Kappa and Gwet’s AC1. Therefore, Fleiss’ Kappa was used to interpret the findings. The agreement levels between two or three DDI databases were generally low (i.e. 0.277 between Pharmavista and MediQ and 0.131 for the three databases), echoing earlier findings by Amkreutz et al., with a concordance coefficient of 0.23 [95% CI 0.11, 0.35] between Pharmavista and MediQ for DDIs focusing on immunosuppressants (Amkreutz et al., 2017). Similarly, Sheriff et al. reported a low level of consistency (0.174 [95% CI 0.145, 0.203]) across eight interaction databases, including Lexi-Interact, for the most commonly used drug pairs in the United Arab Emirates (Shariff et al., 2021). To our knowledge, no studies have been published that specifically compare DDI databases for oral anticoagulants.

Regarding high-risk DDIs, only 5.3% and 1.5% were common among the three databases for DOAC and VKA, respectively, which agrees with the literature (ThomasFulda et al., 2000; Abarca et al., 2004; Wong et al., 2008; Wang et al., 2010). A critical appraisal of the discrepancies showed that for PK DDIs, mostly inconsistencies arose on one hand from the uncertainty in the mechanism of the DDI due to the lack of informative PK data (e.g., chloral hydrate, orlistat) and on the other hand from the lack of robust information in the literature of the inhibitory or inducing potency of the interactor involved. According to the references used, a drug can indeed be classified as a strong, moderate or low CYP inhibitor, which influences as a consequence the risk classification. As an example, dronedarone is classified as high-risk by Pharmavista, moderate by Lexi-Interact and low risk by MediQ. Furthermore, in some cases, the risk associated with the molecule was not directly attributable to its own pharmacological properties, but rather to its combination or concomitant use with other molecules. This could explain some differences in classification for drugs associated by default to the same risk level of others of molecules of the same therapeutic class (e.g., antifungal agents) or for those always co-administered with pharmacokinetic boosters, but without intrinsic interaction (lopinavir w/ritonavir). For high-risk pharmacodynamic DDIs, unclear mechanisms of action contributed to varied classifications among the databases. Pharmavista, opting for a precautionary approach, tended to categorize uncertain mechanisms as severe due to limited information, as observed for example with the interaction between protein kinase inhibitors and a DOAC. Another point of divergence was the consideration of adding two anticoagulants together. While co-administration of two VKAs or two DOACs is clinically contraindicated, MediQ included such interactions in its database, while Pharmavista chose to omit them, considering it clinically implausible. Lexi-Interact regarded it as a common strategy during a VKA switch, though typically, switches occur between a DOAC and a VKA, not between two VKAs. Additionally, the underlying mechanism of varied for some drugs; For example, Lexi-Interact attributes the tipranavir-VKA interaction to an additional inhibition of platelet aggregation, whereas Pharmavista suggests probable Pgp inhibition.

Globally, there are several reasons explaining the discrepancies among the DDI databases. Firstly, each database adopts different criteria for the inclusion of DDI; some encompass all molecules within a therapeutic class, while others are more selective. Secondly, the references used to guide the classification of the severity scores do not seem to be homogeneous between the databases (Kongsholm et al., 2015), as a consequence of a mismatch between the information provided by the review of primary and secondary literature, unpublished data released by pharmaceutical companies, product labels, and reports collected by post-marketing surveillance reports. Thirdly, the lack of *in vitro* or *in vivo* data amplifies the heterogeneity of the knowledge of the nature, mechanism and potency of DDIs. Of particular concern is the lack of agreement between sources in the potency of CYP-mediated interactions. Such discrepancies can have significant clinical implications, as accurate knowledge of CYP-mediated interactions is very important for adjusting drug dosages and avoiding potential adverse reactions. Furthermore, the various classification levels of each database complexify the comparison and the interpretation of the provided information (Scheife et al., 2015). These discrepancies could be problematic in clinical practice, affecting decision-making (Paterno et al., 2009) and patient safety. This can lead to a loss of confidence in clinical decision support tools that incorporate DDI databases, and lead to different treatment decisions, depending on which database the healthcare professional relies on. It is important to recognize that DDIs constitute only one risk factor among several that may influence the PK and PD of oral anticoagulants. Additional risk factors include co-morbidities that can lead to DDIs with oral anticoagulants, such as hepatic and renal impairment (Chang et al., 2021; Canonico et al., 2022).

Our study highlighted a lack of transparency in several areas: the data extraction process, the methods used to categorize the severity of interactions, and potential conflicts of interest. Information was presented inconsistently across the DDI databases. Lexi-Interact was the only platform that provided its standard operating protocol for DDI severity categorization and defined a level of evidence for the information used to classify DDIs, setting a higher standard of transparency in this regard. Pharmavista was the only database to explain its data extraction process on its website, as it exercises control over the production of the expert information provided by manufacturers, further enhancing the transparency of its operations and providing insight into its data collection procedures. In a unique position, MediQ explicitly mentioned staff qualifications on its website, providing a clearer picture of the expertise involved in database management. However, despite these positive aspects, none of the databases provided information on potential conflicts of interest, highlighting an area where further transparency and disclosure could be beneficial in ensuring the integrity of the data and information provided to healthcare professionals and researchers. Our findings are corroborated by results from a systematic review conducted by Kongsholm et al. which assessed the online transparency regarding ownership, funding, information provision, classifications, staff training, and foundational documentation for eight databases (including Lexi-Interact). This review also highlighted the variations in content between databases and emphasized the importance of transparency in the processes of extraction, classification and standardization of the terminology used to classify DDIs (Kongsholm et al., 2015).This lack of transparency causes a black box effect and may prevent the prescriber from positioning himself correctly (Kongsholm et al., 2015), although suggestions have been made to standardize the assessment of evidence and clinical consequences (Far et al., 2012; Hines et al., 2012; Payne et al., 2015; Scheife et al., 2015).

These findings highlight the necessity for the clinical practitioner to cross-reference several sources of information. However, in clinical practice, time constraints often challenge the feasibility of such comprehensive cross-referencing. Several recommendations and strategies can be considered to improve the consistency and accuracy of drug interaction information (DDI) in online databases: the elaboration of a unique risk classification scale in order to help healthcare professionals and researchers to interpret the severity of DDIs in a consistent manner (Smithburger et al., 2011), an individual drug-based classification to minimize the risk of including in a risk classification drugs that have different class effects, and a collaborative data integration by combining data from multiple databases, involving experts groups and clinical experience to improve the congruence of DDI severity assessments through expert consensus classification (Smithburger et al., 2010; Smithburger et al., 2011). Our results suggest that a more specific DDI tool for oral anticoagulants should be developed, transparent in its approach for extraction and classification of the DDIs to enhance safe drug prescription of these high-risk drugs. Strategies have now been developed to help clinicians make decisions about DDIs with this therapeutic class. For example, consider switching to a VKA when a CYP3A4 or Pgp inhibitor leads to a doubling of the exposure (AUC) of a DOAC, or when an inducer leads to a reduction of at least 20% in its AUC (Steffel et al., 2021; Terrier et al., 2021). It is essential to target more accurately the DDIs that require action, to minimize the “over-alerting” effect commonly encountered with tools embedded in Clinical Decision Support Systems (CDSS).

Some limitations of our study should be addressed. To begin, the absence of a gold-standard limited the evaluation of each database’s risk categorization adequacy. In addition, the merge of the proposed risk levels into three equivalent risk level (high, moderate and low) might have increased the number of mismatches between the three databases. The use of uniform severity rating scales, as previously suggested, should be more widely accepted to simplify the comparisons (Amkreutz et al., 2017; Shariff et al., 2021; Kontsioti et al., 2022). We focused on the three databases mostly used in Switzerland, which could be considered as a limitation. However, our results are congruent with previous studies and the main message of a lack of concordance in the risk classification among software appears to be generalizable. Also, the absence of warfarin information that is not available on the Swiss market is a limitation. Yet, its potential for DDI is close to that of phenprocoumone, which limits this bias. Antithrombotics are at the forefront of the risk of DDIs. DOACs often show in real-world settings significant variations in dose-concentration response due to DDIs (Vazquez, 2018; Foerster et al., 2020). The clinical impact of inconsistent DDI information for these drugs as revealed by our analysis can be major, especially considering that antithrombotics are among the drugs with the highest risk of adverse events (Lee et al., 2020). Our research explored the complexity of these antithrombotic DDIs and the challenges of transparency in DDI databases. We found notable inconsistencies and gaps in transparency across the major drug databases, creating a “black box effect.” While some databases, such as Lexi-Interact, Pharmavista and MediQ, offer some degree of transparency, there is a glaring lack of consistency. This lack of consistency, particularly for oral anticoagulants, suggests an urgent need for a refined, transparent DDI tool tailored to these drugs. Our findings, novel in the comparison of DDI involving oral anticoagulants across three widely used clinical practice DDI databases, underscore the importance of providing clinicians with clear, reliable DDI information to ensure safe prescribing and patient health.

## 5 Conclusion

Preventing DDIs related to oral anticoagulants is of major relevance for ensuring patient safety. This study highlights the discrepancies between three commonly used DDI databases and the inconsistency in how terminology is standardized and incorporated when classifying these DDIs. The elaboration of a unique risk classification scale, an individual drug-based classification as well as a collaborative data integration combining data from multiple databases with the involvement of expert groups and clinical experience can improve consistency between all DDI database. Our findings emphasize the necessity of creating specialized tools for anticoagulant-related interactions. Such tools could improve the accuracy and clinical relevance of DDI alerts, thereby enhancing patient safety.

## Data Availability

The raw data supporting the conclusion of this article will be made available by the authors, without undue reservation.
