# Supplementary material for: Drug-drug interactions with oral anticoagulants: information consistency assessment of three commonly used online drug interactions databases in Switzerland
Source: Front Pharmacol. 2024 Apr 2;15:1332147. doi: 10.3389/fphar.2024.1332147 (PMC11022661; doi:10.3389/fphar.2024.1332147)
Supplement: Supplementary file 1 [file Table1.docx]

Supplementary Material

# Supplementary Tables

**Supplementary Table A:** Description of DDI across the 3 databases

|  | Classification | **Apixaban** n (%)* | **Rivaroxaban**  n (%)* | **Edoxaban**  n (%)* | **Dabigatran** n (%)* | **Acenocoumarol** n (%)* | **Phenprocoumon**  n (%)* |
| --- | --- | --- | --- | --- | --- | --- | --- |
| **Lexi-Interact** | X | 29 (8.7) | 36 (10.1) | 20 (7.0) | 31 (9.8) | 8 (1.4) | 9 (1.5) |
|  | D | 52 (15.5) | 47 (13.2) | 108 (37.9) | 52 (16.5) | 152 (25.7) | 89 (14.5) |
|  | C | 141 (42.1) | 131 (36.8) | 117 (41.1) | 134 (42.4) | 293 (49.5) | 328 (53.5) |
|  | B | 47 (14.1) | 46 (12.9) | 0 (0.0) | 17 (5.4) | 14 (2.4) | 12 (2.0) |
|  | A | 0 (0.0) | 0 (0.0) | 0 (0.0) | 0 (0.0) | 1 (0.17) | 0 (0.0) |
| **Pharmavista** | 1 | 17 (5.1) | 19 (5.3) | 17 (6.0) | 32 (10.1) | 10 (1.7) | 10 (1.6) |
|  | 2 | 16 (4.8) | 18 (5.1) | 4 (1.4) | 11 (3.5) | 14 (2.4) | 14 (2.3) |
|  | 3 | 44 (13.2) | 50 (14.0) | 41 (14.4) | 20 (6.3) | 134 (22.6) | 137 (22.4) |
|  | 4 | 0 (0.0) | 1 (0.9) | 0 (0.0) | 0 (0.0) | 9 (1.5) | 3 (0.49) |
|  | 5 | 20 (6.0) | 19 (5.3) | 24 (8.4) | 83 (26.3) | 127 (21.5) | 133 (21.7) |
|  | 6 | 0 (0.0) | 0 (0.0) | 0 (0.0) | 0 (0.0) | 3 (0.51) | 3 (0.49) |
| **MediQ** | 3 | 5 (1.5) | 12 (3.4) | 8 (2.8) | 13 (4.1) | 3 (0.51) | 9 (1.5) |
|  | 2 | 40 (12.0) | 26 (7.3) | 16 (5.6) | 33 (10.4) | 64 (10.8) | 100 (16.3) |
|  | 1 | 56 (16.7) | 89 (25.0) | 33 (11.5) | 35 (11.1) | 75 (12.7) | 107 (17.5) |
|  | ? | 6 (1.8) | 9 (2.5) | 7 (2.4) | 6 (1.9) | 15 (2.5) | 14 (2.3) |

**Percentages are based on the total number of DDI for each oral anticoagulant. Lexi-Interact drug-drug information classification: X: Avoid Combination, D: Consider therapy modification, C: Monitor therapy, B: No action needed, A: No known interaction. Pharmavista drug-drug information classification: 1: Serious consequences likely - Contraindicated, 2: Not recommanded or adaptation required, 3: Monitoring required, 4: Monitoring and/or adaptation necessary in some cases, 5: Monitoring (as a precaution), 6: Generally no action required. MediQ drug-drug information classification: 3: High interaction, 2: Average interaction, 1: Low interaction, ?: No interaction.*

**Supplementary Table B** Description of the divergent DDI mechanism for the 16 drugs across the 3 databases

|  | | **Interactor** | **Lexi-Interact** | **Pharmavista** | **MediQ** |
| --- | --- | --- | --- | --- | --- |
| **Apixaban** | Tipranavir | | Decreased platelet aggregation | CYP3A4 and/or Pgp inhibition | No DDI detected |
|  | Azithromycine | | Potential Pgp inhibition | No DDI detected | Potential liver toxicity and thrombocytopenia |
| **Rivaroxaban** | Carvedilol | | Pgp inhibition | No DDI detected | Increased hypotensive effect |
|  | Levetiracetam | | CYP 3A4 induction | No DDI detected | Thrombocytopenia |
| **Acenocoumarol** | Celecoxib | | Unknown mechanism | Increased risk of gastrointestinal ulcers | No DDI detected |
|  | Disulfiram | | CYP 2C9 and 1A2 inhibition | Unknown mechanism | Unknown mechanism |
|  | Doxycycline | | Unknown mechanism. Potential reduction in prothrombin activity or reduction in GI flora (which allows vitamin K production). | No DDI detected | Additive effects |
|  | Tamoxifen | | CYP 2C9 inhibition | Unknown mechanism | Unknown mechanism |
|  | Quinidine | | Unknown mechanism | Anti-vitamin K, hypoprothrombinemic and thrombocytopenic effect. | No DDI detected |
| **Phenprocoumon** | Ceritinib | | CYP 2C9 inhibition | CYP 3A4 inhibition | No DDI detected |
|  | Chloramphenicol | | CYP 2C9 inhibition | CYP 3A4 inhibition | No DDI detected |
|  | Eplerenone | | Decreased anticoagulant effect - unknown mechanism | No DDI detected | Increased hypoglycaemic effect |
|  | Fusidic Acid | | Potential inhibitor of CYP 3A4 (controversial). | No DDI detected | Liver function disorders: impaired formation of coagulation factors. |
|  | Ifosfamide | | Potential CYP 3A4 inhibitor | Potential CYP 2C9 inhibition | No DDI detected |
|  | Oxacillin | | Unknown mechanism but potential eradication of GI flora (which allows vitamin K production), or preferential hepatic metabolism of clavulanate over warfarin: ↑ [warfarin]. | Potential induction of metabolism | No DDI detected |
|  | Tamoxifen | | CYP 2C9 inhibition | Unknown mechanism | Increased inhibition of blood coagulation |

*CYP: Cytochrome P450, Pgp: P-glycoprotein, DDI: Drug-drug interaction.*

**Supplementary Table C**: Information details concerning the drug-drug interaction databases.

| Information type | Information details | | |
| --- | --- | --- | --- |
| Database | Lexi-Interact | Pharmavista | MediQ |
| Ownership | | | |
| Formal ownership | Provided by Wolthers Kluwer Health. | Provided by HCI solutions SA | The Aargau Psychiatric society |
| Extraction and classification of interactions | | | |
| Presence of a severity classification | X = Avoid Combination D= Consider therapy modification C= Monitor therapy B= No action needed  A= No known interaction | 1. Serious consequences likely - Contraindicated  2. Not recommanded or adaptation required  3. Monitoring required  4. Monitoring and/or adaptation necessary in some cases  5. Monitoring (as a precaution) 6. Generally no action required | U= Unusual combination Red= High interaction Orange = Average interaction Yellow= Low interaction  Grey = No interaction |
| Standard operating procedure for categorization of severity | Yes | No | No |
| Level of evidence defined | Excellent Good Fair Poor | No | No |
| Standard operating procedure for categorization of level of evidence | A. Excellent: The interaction is supported by 2 or more well-conducted, well-controlled human studies. Evidence for an interaction greatly outweighs evidence against an  interaction.  B. Good: The interaction is supported by at least one well-conducted, well-controlled human study or at least 3 cases and/or lower-quality studies. C. Fair: The interaction is supported by at least one of the following: at least 2 cases; a human study; evidence that would otherwise qualify for a higher documentation rating but for which there are substantial contradictory results or reports; product labelling statements not supported by additional evidence. Conflicting data may exist, but evidence of no interaction is not greater than evidence of an interaction.  D. Poor: The interaction is supported by a single case report or when substantial contradictory results or reports exist and evidence against an interaction greatly outweighs evidence for an interaction. | No | No |
| References available for each interaction | Yes | Yes | Yes |
| Presence of clinical recommendations | Yes | Yes | Yes |
| Primary information sources | | | |
| Description of primary sources used | Primary scientific literature published in PubMed journals, and manufacturer prescribing information. Secondary and tertiary literature (e.g., clinical guidelines, systematic reviews) are primarily used for supporting documentation. | Based on professional information recommendations (mainly Swiss, but also foreign, e.g., via EMA or FDA). Literature search or consultation of other specialized sources, e.g., crediblemeds.org, especially when professional information is unclear or specific questions arise. | The database has been a medical device since 2020 and is based on specialized information from manufacturers, specialist literature and scientific publications. The data is interpreted and entered according to the clinical experience of their scientific staff. |
| Other | | | |
| Disponibility | Subscription (by paying) | Subscription (by paying) | Subscription (by paying) |
| Information on database updates | Every two weeks (or more often as appropriate with new drugs that are immediately available with significant interaction  considerations or updates of particular public health importance). | Presence of the date of the last update of the site, and the date of the text explaining the interaction. | A consistency check is carried out on the database at **regular intervals**. It is checked whether the relations linked at the time of storage, such as comment to active substance or drug to active substance, are still consistent. If an inconsistency is found, the licensed users are no longer allowed on the system until the inconsistency is resolved. |
